# Editorial: Improving outcomes in autism spectrum disorders for adults

**DOI:** 10.3389/fpsyt.2025.1615757

**Published:** 2025-05-12

**Authors:** Robert L. Hendren, Felicia Widjaja

**Affiliations:** Department of Psychiatry and Behavioral Science, University of California, San Francisco, San Francisco, CA, United States

**Keywords:** autism, outcome, adults, review, guidance

Autism Spectrum Disorder (ASD) diagnosis rates increased substantially between 2011 and 2022, particularly among young adults. Data from 12,264,003 members seeking care from one of 12 sites of the Mental Health Research Network (MHRN) found the greatest relative increase in diagnosis rate occurred among 26-to-34-year-olds (450% increase) (1).

Parents of young children diagnosed with autism spectrum disorder (ASD) desperately search for a cure for their child’s disorder. As their child ages, depending on their child’s severity or lack of progress, parents shift from looking for a cure to finding arrangements where their child can live a fulfilling life as an adult when they are less involved and less able to help. Unfortunately, they too often find that the resources they have fought for their children to receive disappear when their youngsters are no longer part of the school support system.

Diagnosing ASD in adults presents the additional developmental challenges of age and symptom presentation and additional challenges such as the acquisition of learnt or camouflaging strategies. There is a high frequency of co-occurring disorders. Misdiagnosis is possible, especially in women (2).

Outcomes reported in adults with ASD are concerning. Young adults with ASD have the lowest employment rate across disabilities. Odds of ever having had a paid job were higher for those who were older, white, from higher-income households, and with better conversational abilities, functional skills, and work experience (3).

Proceedings from a capacity-building conference in Vancouver, Canada found that autistic adults report access, anxiety/depression, GI, and sleep the most important issues. Struggles for patients and families included insufficient regional clarity on guiding principles for navigation services, a lack of resources relative to family need for services, and insufficient infrastructural supports in regions (4).

In this Special Section, the first manuscript, “Profile and development of adaptive behavior in adults with autism spectrum disorder and severe intellectual disability (Adrien et al.), reports the relationships between the levels of the different domains and subdomains of adaptive development and the intensity of autistic symptomatology. Results from the Vineland and other measures are reported for seventy-one adults with ASD, severe ID, somatic problems (epilepsy or genetic syndrome), and behavioral difficulties. The participants were recruited from several medico-social institutes in six French regions and the recruitment was empirically based on spontaneous contacts.

Among the nine sub-domains, the weakest developmental levels corresponded to Expressive Language, Interpersonal Relationships, and Play/Leisure. Higher abilities were reported in receptive than in expressive communication Their socialization skills were lacking in terms of interpersonal relationships and the management of play and leisure. On the other hand, they showed more social adaptation skills with familiar people.

The second paper in this section is on depressive symptoms in 315 autistic adults (Zheng et al.) provided from five waves of data over 2 years on self-reported depressive symptoms and sociodemographic and life circumstances information.

Autistic adults with a depression history and lower annual household income reported higher levels of depressive symptoms. Notably, autistic adults reported lower depressive symptoms when they were engaged in work or school. The autistic adults with a depression history started with a higher level of depressive symptoms and remained persistently at elevated levels over the two-year study period. The findings highlight the value of examining the differential impact of key mental health factors in autistic adults and their socioeconomic background.

The potential importance of targeting circadian rhythms in the management and treatment of ASD is highlighted in the third paper (Zhang et al.). The potential role of circadian dysfunction in neurodevelopmental phenotypes in ASD is reviewed as is its role as a target or modulator in the management of ASD, such as phototherapy, melatonin, modulating circadian components, natural compounds, and chronotherapies. Novel strategies for enhancing ASD treatment are explored.

The fourth paper in this section explores the health information needs of individuals with autism spectrum disorder (ASD) and their caregivers by analyzing discussions from Reddit (Larnyo et al.). The timeframe of COVID-19 was selected to capture the evolving health information needs of autistic individuals in the context of the pandemic’s impact. The results were reported for 232 users and their respective posts during this timeframe. The themes found ranged from symptom descriptions and diagnostic challenges to treatment options, caregivers and the need to address stigma.

The final manuscript in this section (Abouzed et al.) describes the sleep quality of 83 individuals with ASD, between 8 and 25 years using the Pittsburgh Sleep Quality Index. Screen time and pre-bedtime technology use was very significantly associated with poorer sleep quality. The discussion suggests the association between sleep quality and the use of smart technology may be even stronger for autistic individuals.

The five papers in this section address improving outcomes in adolescents and adults with ASD and add significantly to the small but growing literature in this area. The complexity of age, developmental and functional level, comorbidity, and resources among others add to the need for further studies of outcome for autistic adults and their families.

Meaningful outcomes for the person with ASD and his or her family varies depending upon the complex definition of “meaningful”. Although education and employment are often chosen as “good outcomes,” they may not be the most meaningful for families or individuals with ASD who identify such outcomes as friendships, having a place to live, and health of family members as the most meaningful. Differences between male and female individuals, diversity, developmental level, socialization and loneliness, and the success of community partnerships to bridge research to clinical practice can all affect what is considered meaningful (5).
